# Lung diffusing capacity for nitric oxide and carbon monoxide in relation to morphological changes as assessed by computed tomography in patients with cystic fibrosis

**DOI:** 10.1186/1471-2466-9-30

**Published:** 2009-06-16

**Authors:** Holger Dressel, Laura Filser, Rainald Fischer, Katharina Marten, Ullrich Müller-Lisse, Dorothea de la Motte, Dennis Nowak, Rudolf M Huber, Rudolf A Jörres

**Affiliations:** 1 Institute and Outpatient Clinic for Occupational, Social and Environmental Medicine, Ludwig-Maximilians-University, Ziemssenstr. 1, D-80336 München, Germany; 2 Department of Pneumology, Medizinische Klinik Innenstadt, Ludwig-Maximilians-University, Munich, Germany; 3 Department of Diagnostic Radiology, University of Göttingen, Göttingen, Germany; 4 Institute for Clinical Radiology, Medizinische Klinik Innenstadt, Ludwig-Maximilians-University, Munich, Germany

## Abstract

**Background:**

Due to large-scale destruction, changes in membrane diffusion (Dm) may occur in cystic fibrosis (CF), in correspondence to alterations observed by computed tomography (CT). Dm can be easily quantified via the diffusing capacity for nitric oxide (DL_NO_), as opposed to the conventional diffusing capacity for carbon monoxide (DL_CO_). We thus studied the relationship between DL_NO _as well as DL_CO _and a CF-specific CT score in patients with stable CF.

**Methods:**

Simultaneous single-breath determinations of DL_NO _and DL_CO _were performed in 21 CF patients (mean ± SD age 35 ± 9 y, FEV_1 _66 ± 28%pred). Patients also underwent spirometry and bodyplethysmography. CT scans were evaluated via the Brody score and rank correlations (r_S_) with z-scores of functional measures were computed.

**Results:**

CT scores correlated best with DL_NO _(r_S _= -0.83; p < 0.001). Scores were also related to the volume-specific NO transfer coefficient (KNO; r_S _= -0.63; p < 0.01) and to DL_CO _(r_S _= -0.79; p < 0.001) but not KCO. Z-scores for DL_NO _were significantly lower than for DL_CO _(p < 0.001). Correlations with spirometric (e.g., FEV_1_, IVC) or bodyplethysmographic (e.g., SR_aw_, RV/TLC) indices were weaker than for DL_NO _or DL_CO _but most of them were also significant (p < 0.05 each).

**Conclusion:**

In this cross sectional study in patients with CF, DL_NO _and DL_CO _reflected CT-morphological alterations of the lung better than other measures. Thus the combined diffusing capacity for NO and CO may play a future role for the non-invasive, functional assessment of structural alterations of the lung in CF.

## Background

Cystic fibrosis (CF) is characterized by the combined findings of bronchiectasis and parenchymal fibrosis, which may affect lung diffusing capacity. However, the results of studies focussing on the diffusing capacity for carbon monoxide (DL_CO_) in CF are largely contradictory, as a spectrum of elevated, decreased, or normal values has been reported [1,2]. Recent data in children and adults suggest a slightly elevated DL_CO _in early CF and a reduction only in advanced disease [3-5]. Based on these findings, DL_CO _currently appears to play no role in CF assessment compared to spirometry or computed tomography (CT) [6].

The diffusing capacity for nitric oxide (DL_NO_) can be used to directly describe pulmonary membrane diffusing capacity (Dm), without interfering with pulmonary capillary blood volume (Vc). Diffusion properties of NO are similar to those of CO; however, its rate of reaction with red blood cells is much greater [7]. DL_NO _primarily reflects Dm, whereas DL_CO _depends on both Dm and Vc [7]. In combination with DL_CO_, Vc and Dm can be determined in a single maneuver, based on the equation for the serial connection of resistances [8,9].

Thus, DL_NO _might be superior to DL_CO _for quantification of structural changes by purely functional means when morphological changes of gas transport compartments instead of changes in pulmonary blood volume are considered. The method of choice for the assessment of morphological changes in CF is thin-section CT [10], which also represents the gold standard for the diagnosis of bronchiectasis [11]. Quantification of disease extent in patients with CF can be achieved using dedicated scoring systems focussing on different parameters, e.g. bronchiectasis, mucous plugging or bronchial wall thickening [12]. Specifically, the CT scoring system developed by Brody et al. has been shown to be a robust and reproducible tool for the semiquantitative assessment of parenchymal and airway disease in CF patients [13].

The aim of our study was to correlate DL_NO_, DL_CO _and other pulmonary function analyses with the extent of disease on CT in order to further investigate the hypothesis that DL_NO _as a measure of Dm may accurately reflect morphological changes in patients with CF.

## Methods

### Study subjects

Adult patients with CF (n = 21) in whom thin-section CTs of the lungs were available were recruited from the Cystic Fibrosis Outpatient Unit during routine follow-up visits. Patients with a smoking history or with an interval of more than 3 years between CT scans and pulmonary function tests (PFT) were excluded from the study. During the time between the CT and the PFT the clinical status of the patients had been regularly monitored by specialists from the Cystic Fibrosis Outpatient unit, usually with 3 months intervals between visits. Patients were excluded if severe exacerbations were present when CT scans or PFTs were performed. They were also excluded if severe exacerbations were recorded during the time interval between CT and PFT. Any exacerbation resulting in one or more days of hospitalisation was defined as severe. The study was approved by the local Ethics Committee and all participants gave their written informed consent.

### Measurements

Simultaneous single-breath determinations of DL_CO_, DL_NO _and alveolar volume (VA), were performed with a Masterscreen PFT (Viasys/Jaeger, Höchberg, Germany) which uses electrochemical sensors at an inspiratory target concentration of 45 (± 5) ppm NO and a breath-hold time of 8 s [14]. Patients were seated in upright position, wearing a nose clip. The final inspiratory gas (21% O_2_) was mixed from a gas containing 448 ppm NO in N_2 _(Linde, Unterschleiβheim, Germany), a mixture of 0.28% CO and 9.5% He in synthetic air (Viasys), and 100% oxygen and analyzed immediately before inhalation. Maneuvers were performed as described previously [8] and the device was calibrated at least daily. Hb was measured in the patients and DL_CO _was corrected to the standard Hb according to international guidelines [8]. All measurements were performed threefold with an interval of approx. 4 min, and mean values were recorded. Values relative to volume (KNO, KCO) were obtained by dividing DL_NO _and DL_CO _through the corresponding VA. VA, DL_NO _and DL_CO _%predicted were calculated using published reference formulae [15-19]. Using a standard approach Dm was expressed as DL_NO _divided by 1.97 [18], and Vc was derived as previously described [9].

Spirometry and bodyplethysmography were performed using a pneumotachograph-based device (MasterLab™, Viasys) following established guidelines [20]. At least three technically acceptable flow-volume curves were obtained and the highest values of forced expiratory volume in 1 second (FEV_1_) and inspiratory vital capacity (IVC) were recorded. Airway resistance (R_AW_), specific airway resistance (SR_AW_), intrathoracic gas volume (ITGV), total lung capacity (TLC) and residual volume (RV) were also obtained.

### CT examinations and scoring

As CF patients in this clinic are usually monitored by CT every 3 years we used the most recent high resolution CT scans obtained for routine follow-up within this period. Among the 21 CTs, 15 had been performed on the same day as the measurements or within the last 12 months (mean ± SD interval: 10 ± 10 months, range: 0–33 months).

Unenhanced low-dose CT examinations were performed on a clinical whole-body multidetector-row CT scanner (Mx 8000, Philips Medical Systems, Best, The Netherlands), with the patient in supine position, at 120 kV, 4 × 1 mm slice collimation, 3 mm reconstruction slice thickness, 10 mAs/slice (effective tube current-time product), and Pitch 1.75, resulting in an effective dose of approximately 0.5 mSv. Multiplanar image reformatting was performed in all instances, such that axial, coronal, and sagittal CT images were available for review on a picture archiving and communication system (PACS, "Impax", AGFA, Munich, Germany). CT images were evaluated by two independent experienced chest radiologists blinded to clinical or functional information, using a validated scoring system [13]. The order in which CTs were scored was not randomized. In detail, scores for the presence and severity of parenchymal findings and airway disease were calculated of each lobe, with the lingula counted as a separate lobe. The following subscores were obtained by averaging subscores across the 6 lobes in each patient: bronchiectasis, mucous plugging, peribronchial thickening, and parenchymal disease. As expiratory scans were not available, the air trapping subscore was omitted. The scoring system has been described in detail by Brody at al. [13]. In brief, the subscore for bronchiectasis (possible range: 0–12) was defined as the sum of the extent of bronchiectasis in the central lung (0–3) and in the peripheral lung (0–3), multiplied by the average bronchiectasis size (0–2). For the mucous plugging score (0–6) the extent of mucous plugging in the central lung (0–3) was added to the extent of mucous plugging in the peripheral lung (0–3). For the peribronchial thickening score (0–9) the sum of the extent of peribronchial thickening in the central lung (0–3) and the extent of peribronchial thickening in the peripheral lung (0–3) was multiplied by the severity of peribronchial thickening (1–1.5). The parenchyma score (0–9) was the sum of the extent of dense parenchymal opacity (0–3), the extent of ground glass opacity (0–3) and the extent of cysts or bullae (0–3). Subscores were added to obtain the total score, ranging between 0 and 36. The mean scores of both observers were used for analysis.

### Statistical analysis

Median with range or mean values and standard deviations (SD) were computed. Z-scores were additionally calculated where appropriate using published equations [15-17,21]. The relationship between functional measures and CT scores was quantified by Spearman's rank correlation (r_S_). Statistical comparisons were performed using the Wilcoxon test. Statistical significance was assumed for p < 0.05. To adjust the alpha level for multiple tests for correlation the Bonferroni method was used. All analyses were performed using SPSS 14.0 (SPSS Inc., Chicago, IL).

## Results

Patient demographics and results of pulmonary function tests are displayed in table 1. Using standard reference equations [16], mean ± SD DL_CO _was 83 ± 18 %pred and the respective z-score ± SD was -1.3 ± 1.4. According to 3 recently published reference value equations using gender, age, and height as predictors, respective mean DL_NO _%pred and DL_CO _%pred for the patient group were: 71 ± 19 and 82 ± 17 %pred [17]; 60 ± 17 and 75 ± 16 %pred [18]; 63 ± 18 and 86 ± 19 %pred [19]. DL_NO _%pred was always lower than DL_CO _%pred (p < 0.001 each). Z-scores for DL_NO_, that could be calcuated from [17] were -2.3 ± 1.5. When comparing DL_NO _and DL_CO _expressed as z-scores according to references [16,17], DL_NO _was significantly lower than DL_CO _(p < 0.001). 13 subjects (62%) had a z-score < -1.96, i.e. below the 2.5^th ^centile concerning DL_NO _compared to only 8 subjects (38%) concerning DL_CO_.

**Table 1:** Basic characteristics and pulmonary function tests of 21 patients (females = 8; males = 13) with CF

	Mean	% Predicted	z-score
Age, yr	35 ± 9	-	-
Height, cm	176 ± 11	-	-
Weight, kg	65.5 ± 13.4	-	-
VA, L	5.45 ± 1.78	87 ± 19	-1.3 ± 2.0
DL_NO_, mmol × min^-1 ^× kPa^-1^	34.7 ± 12.2	71 ± 19	-2.3 ± 1.5
DL_CO_, mmol × min^-1 ^× kPa^-1^	9.1 ± 2.7	83 ± 18	-1.3 ± 1.4
DL_NO_/DL_CO_	3.8 ± 0.4	^§^	^§^
KNO, mmol × min^-1 ^× kPa^-1 ^× L^-1^	6.34 ± 0.91	84.7 ± 11.9	-1.9 ± 1.6
KCO, mmol × min^-1 ^× kPa^-1 ^× L^-1^	1.69 ± 0.18	81.6 ± 7.4	-1.2 ± 0.6
Dm, mmol × min^-1 ^× kPa^-1^	17.6 ± 6.2	^§^	^§^
Vc, mL	80 ± 25	^§^	^§^
FEV_1_, L	2.58 ± 1.35	66 ± 28	-2.7 ± 2.3
IVC, L	4.18 ± 1.56	88 ± 23	-1.1 ± 2.1
FEV_1_/VC, %	59 ± 13	73 ± 15	-3.2 ± 1.8
R_AW_, kPa × s × L^-1^	0.43 ± 0.25	^$^	^$^
SR_AW_, kPa × s	1.97 ± 1.24	^$^	^$^
ITGV, L	4.14 ± 1.10	128 ± 27	1.6 ± 1.6
RV, L	2.90 ± 1.06	162 ± 51	2.9 ± 2.5
TLC, L	7.08 ± 1.69	108 ± 15	0.8 ± 1.4

^§^Not computed due to lack of sufficient and standardised data

^$ ^Not computed, as reference equations are not commonly used for interpretation

Data are presented as mean ± SD. Abbreviations: VA = alveolar volume, DL_NO _= lung diffusing capacity for nitric oxide, DL_CO _= lung diffusing capacity for carbon monoxide, KNO = nitric oxide transfer coefficient, KCO = carbon monoxide transfer coefficient, Dm = membrane diffusing capacity, Vc = pulmonary capillary blood volume, FEV_1 _= forced expiratory volume in 1 s, VC = maximal inspired vital capacity, R_AW _= airway resistance, SR_AW _= specific airway resistance, ITGV = intrathoracic gas volume, RV = residual volume, TLC = total lung capacity. Prediction equations for VA according to [15], for DL_CO_/KCO according to [16], for DL_NO_/KNO according to [17], and for lung volumes according to [21].

The median (range) total CT score in our sample was 10.7 (3.4–26.5). The corresponding subscore values were 3.9 (0.8–11.2) for bronchiectasis, 1.8 (0.6–4.5) for mucous plugging, 4.0 (0.8–8.7) for peribronchial thickening, and 1.3 (0.4–4.0) for the parenchyma subscore. The total CT scores correlated closely between the two observers, as indicated by the high intraclass correlation (ANOVA, R = 0.91), as well as the rank correlation coefficient (r_S _= 0.897; p < 0.001) and pairwise comparison according to Wilcoxon (p = 0.465). Subscores also significantly correlated between both observers (p < 0.001 each), despite differences in bronchiectasis and parenchyma subscores (Wilcoxon, p < 0.01 each).

**Figure 1:**
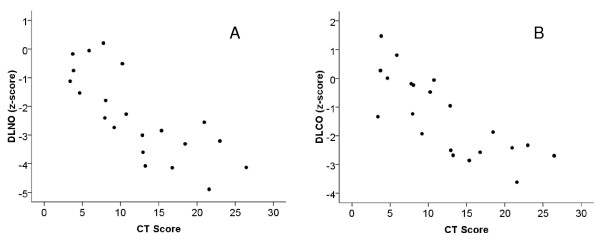
**Scatterplots for z-scores of DL_NO _(Panel A, r_S _= -0.83; p < 0.001), and DL_CO _(panel B, r_S _= -0.79; p < 0.001) against total CT score**.

**Figure 2:**
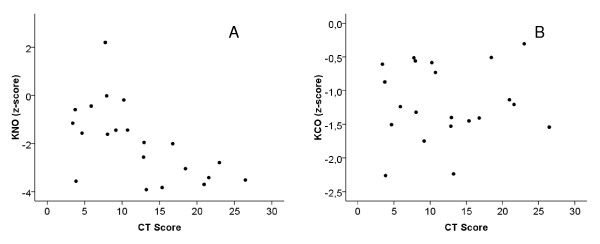
**Scatterplots for z-scores of KNO (Panel A, r_S _= -0.63; p = 0.002) and KCO (Panel B, r_S _= 0.01; n.s.) against total CT score**.

Correlation coefficients between total CT scores and functional measures are given in Table 2. Both DL_NO _and DL_CO_, expressed as z-scores were related to the overall CT score (p < 0.001 each; Figure 1, Panel A and B). Moreover, KNO was correlated with the CT score (p = 0.002), as opposed to KCO (Figure 2, Panel A and B). Similar results as for the total score were obtained for the subscores. DL_NO_, DL_CO_, Dm and Vc showed the closest correlations with bronchiectasis, mucous plugging and peribronchial thickening. With regard to KNO, the correlation was closest for peribronchial thickening, whereas KCO was not related to any of the subscores. The correlations between CT scores and indices of spirometry and bodyplethysmography were generally weaker than with DL_NO _and DL_CO_. When adjusting the alpha level according to Bonferroni for all correlations that were tested, the correlations of both diffusing capacities with the overall CT score, as well with the subscores for bronchiectasis, mucous plugging and peribronchial thickening remained significant. Concerning spirometric and bodyplethysmographic indices the overall CT score as well as the subscores for bronchiectasis and peribronchial thickening remained significantly correlated with SR_AW_.

**Table 2:** Spearman rank coefficients between CT scores according to Brody et al. [13] and z-scores of lung function measures as obtained in bivariate analyses.

	Bronchiectasis	Mucous plugging	Peribronchial thickening	Parenchyma	Overall CT Score
DL_NO_/Dm	-0.78***	-0.73***	-0.79***	-0.60**	-0.83***
DL_CO_	-0.75***	-0.73***	-0.76***	-0.59**	-0.79***
KNO	-0.53*	-0.56**	-0.62**	-0.40	-0.63**
KCO	0.10	0.02	0.03	0.23	0.01
Vc, mL^§^	-0.64**	-0.58**	-0.59**	-0.50*	-0.63**
IVC	-0.50*	-0.51*	-0.54*	-0.50*	-0.55*
FEV_1_	-0.56**	-0.53*	-0.59**	-0.43	-0.59**
SR_AW_, kPa × s^$^	0.69***	0.61**	0.71***	0.61**	0.72***
RV	0.47*	0.43*	0.50*	0.35	0.51*
RV/TLC, %^§^	0.64**	0.58**	0.65**	0.51*	0.66**

^§^No z-scores used due to lack of sufficient and standardised data

^$ ^No z-scores used, as reference equations are not commonly used for interpretation

P values refer to individual pairwise correlations. Correlations remaining significant, when using Bonferroni adjusted alpha levels are indicated in bold. *p < 0.05;**p < 0.01;***p < 0.001, for abbreviations see Table 1

## Discussion

We found that in patients with CF, the correlation between DL_NO _as well as DL_CO _and CT was closer than for indices derived from spirometry and bodyplethysmography, including the standard measure for monitoring CF, FEV_1_. Additionally, using recently published reference values [17-19], DL_NO _%pred was significantly lower than DL_CO _%pred in CF.

At present, spirometry is the established standard in the assessment of patients with CF, while the role of DL_CO _is less well defined. This may be partially explained by the fact that diffusion measurements are influenced by a variety of functional or structural alterations in CF, e.g. the obstruction typically found in those patients, and thus difficult to interpret. In children with CF, DL_CO _has been shown to be stable despite a decline in FEV_1 _%pred [4], and in adults DL_CO _was found to decrease below reference values only in patients with severe disease [3]. Based on these and other conflicting results [2,22] it seems unlikely that DL_CO _is a useful marker of disease severity in CF.

In part, these differences may be due to the fact that DL_CO _essentially comprises two factors: the membrane (Dm) and the blood component (Vc). Changes of these two components do not necessarily correlate. In contrast to DL_CO_, DL_NO _is thought to mainly represent Dm, thus possibly better reflecting morphological alterations in CF. In our study sample values of DL_NO_%pred were regularly significantly lower than those for DL_CO_, using different reference equations [16-19]. This indicates that DL_NO _might be more sensitive for detecting CF-related structural changes of the lung. As different approaches were used to derive Dm and Vc by the authors of the recently published reference equations we did not compare our derived values with these references; further standardization of calculations seems desirable. In this study Dm was calculated as DL_NO_/1.97, whereas recently published data indicates that DL_NO_/2.42 may be more accurate [19]. This however does not change correlations. Dm was more closely related with the CT score than Vc. As DL_CO _consists of Dm and Vc, the strong correlation with the CT score may be explained mainly by changes in Dm. The lower sensitivity to detect CF related changes expressed as percent of predicted values for DL_CO _compared to DL_NO _may be due to the additional influence of the pulmonary capillary blood volume, which is thought to be negligible when measuring DL_NO_. As DL_NO _is thought to be influenced by parenchymal alterations it may complement the standard parameter measuring obstruction, FEV_1_. CT is the standard measurement for structural alterations in CF, usually performed in intervals of several years. As the measurement of DL_NO _is non-invasive, easy to perform and closely correlated with the CT score, it may be applied more often during those routine visits when no CT scans are performed. However, prospective longitudinal studies are needed to decide whether the measurement of DL_NO _may provide additional value to the existing standard monitoring parameters.

As the reduction in diffusion capacities might be caused by a reduction in alveolar volume, which was also correlated with the CT score, we additionally assessed their values relative to alveolar volume. KCO was not related to the CT score or its subscores, while KNO decreased with increasing CT scores (figure 2), being specifically related to bronchiectasis, mucus plugging and peribronchial thickening. With decreasing VA, healthy subjects are known to show an increase in KCO indicating an increase in the thickness of the pulmonary capillary blood sheet [23], while KNO is essentially stable, reflecting the diffusional properties of the lung [17]. Thus, both KCO and KNO reflect different aspects of lung morphology and functionality. In CF, VA and/or TLC may be reduced. This renders KNO, being less related to VA than KCO, superior for a noninvasive assessment of membrane diffusion, indicating structural alterations independent of volume-dependent effects. Our data strongly support this hypothesis. Zavorsky et al. recently published reference equations for DL_NO _using gender and age as well as either VA or height as predictors [19] to discern between patients with abnormal gas exchange or low lung volume. When using the equation including VA with our data mean ± SD predicted DL_NO _was 71 ± 13%, indicating abnormal gas exchange. The equation including height instead of VA resulted in a significantly lower predicted DL_NO _of 63 ± 18% (p < 0.001). According to these authors this means that a reduction in lung volume explains part of the low predicted DL_NO_. This is in line with the finding of a slightly reduced mean VA when expressed as percent of predicted. DL_NO _and DL_CO _also showed stronger correlations with the CT score than measures derived from spirometry and bodyplethysmography. It should be noted that a number of correlation coefficients were rather high, leading to only minor differences. Correlation coefficients were comparable or slightly lower when using absolute values instead of z-score values for DL_NO _and DL_CO_, probably reflecting the fact that lung function indices depend on anthropometric characteristics, in contrast to CT scores.

Among the subscores, the bronchiectasis score correlated best with most functional measures, similar to the total score. Indeed, bronchiectasis seems to be the most important structural change that can be reliably scored on CT [11]. It has even been suggested that restricting scoring to bronchiectasis would suffice for CT monitoring in CF [6]. It should be noted that most subscores reflect airway disease, whereas DL_NO _and KNO are influenced mainly by parenchymal destruction. Thus our findings show a good correlation between a CT score as a marker of disease severity and DL_NO _as a marker of parenchyma destruction. However, a causal connection between single subscores and the changes in diffusing capacities cannot be derived.

Thin-section CT represents the methodology of choice for the assessment of structural alterations in CF [10]. For this purpose, a variety of disease-specific CT scores have been proposed [12] and it seems that further standardization of scores is mandatory. The CT scoring system used in our study has been demonstrated to be reproducible and sensitive to disease severity [13]. CT scores are known to be subject to considerable interobserver variability, and the observation that for selected subscores the two experienced observers differed in their rating is in line with published data [12]. In our study the differences concerned only the magnitude, while ratings were still correlated with each other.

Major weaknesses of this study are its retrospective nature and the time interval between scan and measurements of up to 3 years with a maximum interval of 1 year in 15 patients. Moreover, the order in which CTs were scored was not randomized. This short-coming may introduce additional bias concerning the CT scores.

Recent studies found an annual decline of total CT scores by -2.7% in subjects of an age range from 16 to 48 years [24], or of -1.0% in children and -1.5% in adults [6]. One of those studies report a corresponding decline of FEV_1 _by 2.3% [24] in adults. The other found that the bronchiectasis score deteriorated faster than lung function parameters in children and adults [6]. These findings suggest that the time interval of 3 years did not exert a major influence on our results. Furthermore, only patients with a fairly stable course of their disease were included in our study. It is, however, reasonable to expect even higher correlations with CT scores if these are performed at the same visit as the functional assessments.

## Conclusion

In conclusion, our findings indicated that in patients with CF NO diffusing capacity was a functional measure that was suitable to quantify structural changes of the lung as assessed by CT scores. The suitability of NO diffusing capacity is in line with previous observations in healthy subjects or patients with other diseases than CF [25] and renders it a challenging question of whether the combined diffusing capacity for NO and CO has a potential to be included in the assessment of CF. In this respect, the conclusions of this retrospective cross-sectional study can only be limited. Future longitudinal studies have to decide over the potential of this method for the monitoring of CF patients.

## Competing interests

The authors declare that they have no competing interests.

## Authors' contributions

HD performed analyses and wrote the initial draft of the paper. LF, RF, and DM participated in study design, data collection and interpretation. KM and UM assessed and scored CT scans and drafted parts of the paper. DN and RMH enabled the realisation of the study and participated in data interpretation and drafting the manuscript. RAJ participated in and supervised study design, writing and analysis. All authors read and approved the final manuscript.

## Pre-publication history

The pre-publication history for this paper can be accessed here:


